# Predictors of prolonged hospital stay in a Comprehensive Stroke Unit*

**DOI:** 10.1590/1518-8345.3118.3197

**Published:** 2019-10-14

**Authors:** Ana Paula Gaspari, Elaine Drehmer de Almeida Cruz, Josemar Batista, Francine Taporosky Alpendre, Viviane Zétola, Marcos Christiano Lange

**Affiliations:** 1 Universidade Federal do Paraná, Complexo Hospitalar de Clínicas, Curitiba, PR, Brasil.; 2 Universidade Federal do Paraná, Departamento de Enfermagem, Curitiba, PR, Brasil.; 3 Governo do Estado do Paraná, Secretaria do Estado da Educação, Curitiba, PR, Brasil.

**Keywords:** Stroke, Length of Stay, Patient Discharge, Complications, Hospital Units, Hospital Care, Acidente Vascular Cerebral, Tempo de Internação, Complicações, Alta do Paciente, Unidades Hospitalares, Assistência Hospitalar, Accidente Cerebrovascular, Tiempo de Internación, Complicaciones, Alta del Paciente, Unidades Hospitalarias, Atención Hospitalaria

## Abstract

**Objective:**

to analyze the in-hospital complications of prolonged hospital stay in patients with ischemic stroke or transient ischemic attack, admitted to the stroke unit of a tertiary hospital.

**Method:**

this is an evaluative correlational study. All first-ever ischemic stroke or transient ischemic attack patients admitted were retrospectively analyzed. During hospital stay, the predictors of long-term hospitalization considered were: 1) clinical complications (pneumonia, urinary tract infection, pressure damage and deep vein thrombosis), and 2) neurological complications (malignant ischemic stroke and symptomatic hemorrhagic transformation).

**Results:**

353 patients were discharged in the study period. Mean age was 64.1±13.7 years old and 186 (52.6%) were men. The mean time of hospital stay was 13.7±14.3 days. Pneumonia (25.3±28.8 days, p<0.001), urinary tract infection (32.9±45.2 days, p<0.001) and malignant stroke (29.1±21.4 days, p<0.001) increased significantly the length of hospital stay compared to patients without any complications (11.2±7.1 days).

**Conclusion:**

this study showed that three complications delayed hospital discharge in patients admitted in a stroke unit, two preventable ones: pneumonia and urinary tract infection. More intense measures to avoid them should be included in the performance indicators to reduce the length of hospital stay in stroke units.

## Abstract (pt)

**Objetivo:**

analisar as complicações intra-hospitalares de internação prolongada em pacientes com AVC isquêmico ou ataque isquêmico transitório, internados na unidade de AVC de um hospital terciário.

**Método:**

trata-se de um estudo correlacional avaliativo. Todos os pacientes com primeira incidência de acidente vascular cerebral isquêmico ou ataque isquêmico transitório admitidos foram analisados retrospectivamente. Durante a internação hospitalar, os fatores preditores de internação prolongada foram: 1) complicações clínicas (pneumonia, infecção do trato urinário, lesão por pressão e trombose venosa profunda), e 2) complicações neurológicas (acidente vascular cerebral isquêmico maligno e transformação hemorrágica sintomática).

**Resultados:**

353 pacientes receberam alta no período do estudo. A média de idade foi de 64,1±13,7 anos e 186 (52,6%) eram homens. O tempo médio de internação hospitalar foi de 13,7±14,3 dias. Pneumonia (25,3±28,8 dias, p<0,001), infecção do trato urinário (32,9±45,2 dias, p<0,001) e acidente vascular cerebral maligno (29,1±21,4 dias, p<0,001) aumentaram significativamente o tempo de permanência hospitalar em relação aos pacientes sem quaisquer complicações (11,2±7,1 dias).

**Conclusão:**

este estudo mostrou que três complicações atrasaram a alta hospitalar em pacientes internados em uma unidade de AVC, duas delas evitáveis: pneumonia e infecção do trato urinário. Medidas mais intensas para evitá-las devem ser incluídas nos indicadores de desempenho para reduzir o tempo de permanência hospitalar nas unidades de AVC.

## Abstract (es)

**Objetivo:**

Analizar las complicaciones intrahospitalarias de estancia hopitalaria prolongada en pacientes con accidente cerebrovascular isquémico o accidente isquémico transitorio, ingresados en la unidad de accidente cerebrovascular de un hospital terciario.

**Método:**

Se trata de un estudio evaluativo correlacional. Todos los primeros pacientes con accidente cerebrovascular isquémico o ataque isquémico transitorio ingresados fueron analizados retrospectivamente. Durante la estancia hospitalaria, los factores predictivos de hospitalización prolongada considerados fueron: 1) complicaciones clínicas (neumonía, infección del tracto urinario, daño por presión y trombosis venosa profunda), y 2) complicaciones neurológicas (accidente cerebrovascular isquémico maligno y transformación hemorrágica sintomática).

**Resultados:**

353 pacientes fueron dados de alta en el período de estudio. La edad media fue de 64,1±13,7 años y 186 (52,6%) eran hombres. El tiempo medio de estancia hospitalaria fue de 13,7±14,3 días. La neumonía (25,3±28,8 días, p<0,001), la infección del tracto urinario (32,9±45,2 días, p<0,001) y el accidente cerebrovascular maligno (29,1±21,4 días, p<0,001) aumentado significativamente la duración de la estancia hospitalaria en comparación con los pacientes sin ninguna complicación (11.2±7.1 días).

**Conclusión:**

Este estudio mostró que de las tres complicaciones retrasaron el alta hospitalaria en pacientes ingresados en una unidad de accidente cerebrovascular, dos eran prevenibles: la neumonía y la infección del tracto urinario. Las medidas más intensas para evitarlas deben incluirse en los indicadores de rendimiento para reducir la duración de la estancia hospitalaria en unidades de accidente cerebrovascular.

## Introduction

Stroke is one of the most common causes of mortality and disability with a high impact in the health of world population^1^. In Brazil, although the mortality rate has decreased in the last years, the incidence is still very high^2-3^.

In patients with acute stroke, adequate evaluation and care support during hospitalization are mandatory. The reperfusion therapy, the investigation of the etiological mechanism, the secondary prevention therapy and early rehabilitation could significantly improve the outcome^4-5^. Otherwise, the occurrence of clinical and neurological complications during hospital stay could influence negatively the patient outcome, delaying discharge^6^ and increasing hospital costs^7^ and intra-hospital mortality rates^8^.

In a recent study, 76.9% of patients in a rehabilitation center presented at least one complication related to stroke and 20% had three or more complications^9^. The most common are related to infection, cardiovascular and venous thromboembolism, increasing disability and mortality. The identification of these complication could be considered as triggers of opportunity to improve the procedures and the interventions related to stroke care^10^.

One of the most significant measures introduced in the clinical practice to improve the outcome and to reduce costs and hospital stay was the stroke unit^11-12^: a specific ward where the quality measures can be monitored continually^13^. A previous study demonstrated the impact of those findings in two distinct comprehensive stroke units^14^, showing the value of an organized specialized center, with the nurse team predominantly looking for a better care.

This study aimed to analyze the in-hospital complications that delay hospital discharge in patients with ischemic stroke or transient ischemic attacks (TIA) admitted to the stroke unit of a tertiary hospital, becoming a benchmark to future studies.

## Method

This is an evaluative correlational study. Data from all first-ever ischemic stroke or TIA patients admitted to the Stroke Unit of Hospital de Clínicas (Federal University of Paraná), between October 2012 and September 2015 were retrospectively analyzed.

Inclusion criteria were: patients older than 17 years, with a diagnosis of the first ischemic stroke or TIA. Patients admitted to the stroke unit transferred to an intensive care for supportive management were included. Patients with hemorrhagic stroke or other diagnoses were excluded (seizure, hypoglycemia). The following variables were analyzed: gender, age, arterial hypertension, diabetes mellitus, dyslipidemia, tobacco use, atrial fibrillation, alcoholism, congestive heart failure and coronary arterial disease. During admission, the predictors of long-term stay considered were: 1) clinical complications (pneumonia, urinary tract infection, pressure damage and deep vein thrombosis), and 2) neurological complications (malignant ischemic stroke and symptomatic hemorrhagic transformation).

Analyses were performed using *Statistical Package for the Social Sciences* 20.0 software. Quantitative variables were described by mean and standard deviations, or median with minimum and maximum values. Categorical variables were presented as frequencies and percentages. For comparison of the quantitative variables, Student’s t test, Mann-Whitney or Kruskal-Wallis non-parametric tests were used. Categorical variables were analyzed using a chi-square test or Fisher’s exact test. Normality of data was determined by using the Kolmogorov-Smirnov test. The Spearman’s correlation coefficient was considered to analyze the correlation between two quantitative variables. Statistical significance was accepted for p-values<0.05.

The study was approved by the Institutional Ethics Committee, under Opinion number, 1.891.218.

## Results

A total of 353 patients were discharged in the studied period: 324 (91.8%) with ischemic stroke and 29 (8.2%) patients with TIA. The mean time of hospital stay was 13.7±14.3 days, the mean age was 64.1±13.7 years old, and 186 (52.6%) were men. The median National Institute Health Stroke Scale (NIHSS) on admission was 7 (0 e 29). Table 1 presents the demographic and risk factors of the population. From all the study patients, 130 (36.8%) were submitted to thrombolysis and 15 (78.9%) to decompressive craniectomy secondary to malignant stroke.

**Table 1:** – Demographic and risk factors of patients hospitalized in stroke unit. Curitiba, PR, Brazil, 2017

Variable (n=353)*	n	%
Arterial hypertension	290	82.4
Diabetes mellitus	108	30.6
Hypercholesterolemia	203	57.5
Current smoking	94	26.6
Alcohol intake	35	9.9
Atrial fibrillation	57	16.1
Coronary artery disease	31	8.8
Cardiac heart failure	48	13.6

*It could be more than one risk factor per patient

In-hospital complications occurred in 95 (26.9%) patients. Table 2 demonstrates the length of hospital stay comparing patients with and without complications.

**Table 2:** – Length of hospital stay in patients with and without complications hospitalized in stroke unit. Curitiba, PR, Brazil, 2017

Complications (n=353)*	n	%	Length of hospital stay (mean±sd^†^)	p-value
None	258	73.1	11.2±7.1	
Pneumonia	49	13.9	25.3±28.8	<0.001
Urinary tract infection	17	4.8	32.9±45.2	<0.001
Pressure damage	03	0.8	37.7±20.6	NA^‡^
Malignant ischemic stroke	19	5.8	29.1±21.4	<0.001
Symptomatic hemorrhagic transformation	13	4.0	14.1±9.7	0.638

*It could be more than one complication per patient; ^†^sd = standard deviation; ^‡^NA = not available

## Discussion

The analysis of the length of stay and the study of the performance indicators are important predictors to the management and to improve the hospital care. Length of stay in patients with stroke could be related to many variables, including severity of the stroke, age and comorbidities. In this study, the length of stay had more days if compared to previous studies^1,15-18^, probably because it was done in a comprehensive stroke center, with acute care and rehabilitation process. In relation to age, older patients have higher risk to stroke complications^19^; this study presented similar data compared to previous studies^20^.

This research project showed that two preventable complications—pneumonia and urinary tract infection—and one neurological complication significantly increase the length of hospital stay in a stroke unit after the first-ever ischemic stroke or TIA. Previous studies demonstrated that pneumonia and urinary tract infection were the most common clinical complications after an ischemic stroke, increasing morbidity and mortality^21-23^. The frequency of these complications in the study population was very similar to previous published studies; pneumonia between 10.6% and 21.2%^6,24-25^, and urinary tract infection between 3.2% and 5.0%^6,24^. There is a reciprocal interaction between complications and hospital stay; the infection retards the discharge, and the length of stay increases the risk of infection^26^. In this study, both infections increased in more than two weeks the length of hospital stay.

There are some predictors to pneumonia in patients with stroke, the most common are the severity of the stroke, altered level of consciousness, bronchoaspiration and disability^27^. These will increase the length of stay and hospital costs^28-29^. The early dysphagia diagnosis and management, checking the level of consciousness, and prevention from pulmonary aspiration could reduce the frequency of this critical and preventable event.

Regarding urinary tract infection, recent studies suggest that the absence of urinary catheter had lower risk of infection^30^, this could be related to the technical procedure and the long-term catheter^31^. Monitoring for urinary function, avoiding urinary retention, and prolonged bladder drainage could reduce urinary tract infection^32^. The management of care and preventable procedures could be considered such as aseptic technique and drainage position.

In addition to the clinical complications observed, malignant stroke also increased the length of hospital stay in the current population. Usually, malignant stroke patients need a more intensive supportive care, and specific cases could be submitted to decompressive craniectomy^33^. These patients usually need intensive care support, increasing the length of stay^34^ and requiring more intense rehabilitation program for recovery^35^. Even though most of these reperfusion hemorrhage are asymptomatic, they can sometimes provoke neurologic decline and, when severe, can be fatal^33^. Based on this, the introduction of specific guidelines for the early diagnosis of malignant stroke and the measures to define the outcome in this group of patients should be included in stroke centers^34^, reducing mortality and disability.

Some important limitations of this study are as follows: the data are from a single public teaching hospital in Southern Brazil. As a retrospective study, it is not possible to evaluate if preventive measures were implemented to the prevalent complications, but all patients were admitted to the stroke unit with a standardized management protocol^11^. The results of this research did not include all types of stroke, since patients with hemorrhagic stroke or cerebral venous thrombosis were excluded, not supporting data of those diseases. A last significant point is that patients could extend their stay in hospital to improve the rehabilitation process and to start the secondary prevention therapy.

## Conclusion

This study evidenced that three complications delayed hospital discharge of patients with first-ever ischemic stroke or TIA admitted in a stroke unit, two preventable ones—pneumonia and urinary tract infection. More intense measures to avoid them should be included in the performance indicators to reduce the length of hospital stay in stroke units. These results could be considered as benchmark to future studies.

## References

[1] Feigin VL, Abajobir AA, Abate KH, Abd-Allah F, Abdulle AM, Abera SF (2017). Global, regional, and national burden of neurological disorders
during 1990–2015: a systematic analysis for the Global Burden of Disease
Study 2015. Lancet Neurol.

[2] Lotufo PA, Goulart AC, Passos VMA, Satake FM, Souza MFM, França EB (2017). Cerebrovascular disease in Brazil from 1990 to 2015: Global
Burden of Disease 2015. Rev Bras Epidemiol.

[3] Santana NM, Figueiredo FWS, Lucena DMM, Soares FM, Adami F, Cardoso LCP (2018). The burden of stroke in Brazil in 2016: an analysis of the Global
Burden of Disease study findings. BMC Res Notes.

[4] Ministério da Saúde (BR), Secretaria de Atenção à Saúde, Departamento de Atenção Especializada (2013). Manual de rotinas para atenção ao AVC.

[5] Clarke DJ, Forster A (2015). Improving post-stroke recovery: the role of the multidisciplinary
health care team. J Multidiscip Healthc.

[6] Kasemsap N, Vorasoot N, Kongbunkiat K, Peansukwech U, Tiamkao S, Sawanyawisuth K (2018). Impact of intravenous thrombolysis on length of hospital stay in
cases of acute ischemic stroke. Neuro Psychiatr Dis Treat.

[7] Icagasioglu A, Baklacioglu HS, Mesci E, Yumusakhuylu Y, Murat S, Mesci N (2017). Economic burden of stroke. Turk J Phys Med Rehab.

[8] Alhazzani A, Mahfouz A, Abolyazid A, Awadalla N, Katramiz K, Faraheen A (2018). In Hospital Stroke Mortality: Rates and Determinants in
Southwestern Saudi Arabia. Int J Environ Res Public Health.

[9] Janus-Laszuk B, Mirowska-Guzel D, Sarzynska-Dlugosz I, Czlonkowska A (2017). Effect of medical complications on the after-stroke
rehabilitation outcome. Neuro Rehabil.

[10] Bustamante A, Berrocoso TG, Rodriguez N, Llombart V, Ribó M, Molina C (2016). Ischemic stroke outcome: A review of the influence of post-stroke
complications within the different scenarios of stroke care. Eur J Intern Med.

[11] Lange MC, de Araujo TF, Ferreira LF, Ducci RD, Novak EM, Germiniani FM (2017). Comparing the comprehensive stroke ward versus mixed
rehabilitation ward-the importance of the team in the acute stroke care in a
case-control study. Hospitalist.

[12] Stroke Unit Trialists’ Collaboration (2013). Organised inpatient (stroke unit) care for stroke. Cochrane Database Syst Rev.

[13] Norrving B, Bray BD, Asplund K, Heuschmann P, Langhorne P, Rudd AG (2015). Cross-national key performance measures of the quality of acute
stroke care in Western Europe. Stroke.

[14] Lange MC, Braga GP, Nóvak EM, Harger R, Felippe MJDB, Canever M (2017). Key performance indicators for stroke from the Ministry of Health
of Brazil: benchmarking and indicator parameters. Arq Neuro-Psiquiatr.

[15] Rawla P, Vellipuram A, Khatri R, Maud A, Rodriguez GJ, Cruz-Flores S (2019). Trends in Acute Ischemic Stroke Hospitalizations by Age Groups,
Length of Stay, Mortality and Hospital Costs in the United States From
2000-2014. Stroke.

[16] Asplund K, Sukhova M, Wester P, Stegmayr B (2015). Diagnostic procedures, treatments, and outcomes in stroke
patients admitted to different types of hospitals. Stroke.

[17] Vieira LA, Guedes MVC, Barros AA (2016). Application of glasgow, braden and ranking scales in patients
affected by cerebrovascular accident. J Nurs UFPE.

[18] Moura MC, Casulari LA (2015). The impact of non-thrombolytic management of acute ischemic
stroke in older individuals: the experience of the Federal District,
Brazil. Rev Panam Salud Publica.

[19] Stecker MM, Stecker M, Falotico J (2017). Predictive model of length of stay and discharge destination in
neuroscience admissions. Surgical Neurol Int.

[20] Hauer AJ, Ruigrok YM, Algra A, Van Dijk EJ, Koudstaal PJ, Luijckx GJ (2017). Age–Specific Vascular Risk Factor Profiles According to Stroke
Subtype. J Am Heart Assoc.

[21] Nascimento KG, Chavaglian SRR, Pires PS, Ribeiro SBF, Barbosa MH (2016). Clinical outcomes of ischemic stroke patients after thrombolytic
therapy. Acta Paul Enferm.

[22] Bruening T, Al-Khaled M (2015). Stroke-associate pneumonia in thrombolyzed patients: incidence
and outcome. J Stroke Cerebrovasc Dis.

[23] Conterno LO, Rego CM, Barbosa RWN, Silva CR (2016). Severity of neurological deficit and incidence of nosocomial
infections in elderly patients with acute stroke. Sci Med.

[24] Wang P, Wang Y, Zhao X, Du W, Wang A, Liu G (2016). In-hospital medical complications associated with stroke
recurrence after initial ischemic stroke: A prospective cohort study from
the China National Stroke Registry. Medicine.

[25] Adrees M, Subhanullah, Rasool S, Ahmad N (2017). Frequency of Stroke Associated Pneumonia in Stroke
Patients. APMC.

[26] George AJ, Boehme AK, Siegler JE, Monlezun D, Fowler BD, Shaban A (2013). Hospital-acquired infection underlies poor functional outcome in
patients with prolonged length of stay. ISRN Stroke.

[27] Almeida SRM, Bahia MM, Lima FO, Paschoal IA, Cardoso TAMO, Li LM (2015). Predictors of pneumonia in acute stroke in patients in an
emergency unit. Arq Neuro-Psiquiatr.

[28] Arnold M, Liesirova K, Broeg-Morvay A, Meisterernst J, Schlager M, Mono ML (2016). Dysphagia in acute stroke: incidence, burden and impact on
clinical outcome. PloS One.

[29] Muehlemann N, Jouaneton B, Léotoing L, Chalé JJ, Fernandes J, Kägi G (2019). Hospital costs impact of post ischemic stroke dysphagia: Database
analyses of hospital discharges in France and Switzerland. PloS One.

[30] Sá FM, Fontes CMB, Mondelli AL (2016). Major infections in hospitalized patients with stroke: a
prospective study. Int Arch Medicine.

[31] Labodi LD, Kadari C, Judicael KN, Christian N, Athanase M, Jean K.B (2018). Impact of Medical and Neurological Complications on
Intra-Hospital Mortality of Stroke in a Reference Hospital in Ouagadougou
(Burkina Faso). JAMMR.

[32] Coleman J (2017). Chronic Catheter Associated Complications and Catheter-Associated
Urinary Tract Infection. Pelvic Floor Dysfunct Pelvic Surg Elderly.

[33] Hasan TF, Rabinstein AA, Middlebrooks EH, Haranhalli N, Silliman SL, Meschia JF (2018). Diagnosis and management of acute ischemic stroke. Mayo Clin Proc.

[34] Bongiorni GT, Hockmuller MCJ, Klein C, Antunes ACM (2017). Decompressive craniotomy for the treatment of malignant
infarction of the middle cerebral artery: mortality and
outcome. Arq Neuro-Psiquiatr.

[35] Suyama K, Horie N, Hayashi K, Nagata I (2014). Nationwide survey of decompressive hemicraniectomy for malignant
middle cerebral artery infarction in Japan. Wld Neurosurg.

